# Impact of COVID-19 pandemic on 2-[^18^F]FDG PET/CT imaging work-flow in a single medical institution: comparison among the three Italian waves

**DOI:** 10.1016/j.heliyon.2022.e08819

**Published:** 2022-01-22

**Authors:** Simone Maurea, Claudia Bombace, Ciro Gabriele Mainolfi, Alessandra Annunziata, Ludovica Attanasio, Arnaldo Stanzione, Elide Matano, Brigitta Mucci, Alessandro D'Ambrosio, Claudia Giordano, Mario Petretta, Silvana Del Vecchio, Alberto Cuocolo

**Affiliations:** a Department of Advanced Biomedical Sciences, University of Naples Federico II, 80131, Naples, Italy; b Department of Clinical Medicine and Surgery, University of Naples Federico II, 80131, Naples, Italy; c Department of Diagnostic Imaging, IRCCS SDN, 80142, Naples, Italy

**Keywords:** COVID-19, PET/CT, South Italy

## Abstract

**Purpose:**

To compare the impact of COVID-19 pandemic on 2-[^18^F]FDG PET/CT imaging work-flow during the three waves in a medical institution of southern of Italy.

**Methods:**

We retrospectively reviewed the numbers and results of 2-[^18^F]FDG PET/CT studies acquired during the following three periods of the COVID-19 waves: 1) February 3-April 30, 2020; 2) October 15, 2020–January 15, 2021; and 3) January 18-April 16, 2021.

**Results:**

A total of 861 PET/CT studies in 725 patients (388 men, mean age 64 ± 4 years) was acquired during the three waves of COVID-19 pandemic. The majority (94%) was performed for diagnosis/staging (n = 300) or follow-up (n = 512) of neoplastic diseases. The remaining 49 studies (6%) were acquired for non-oncological patients. The distribution of number and type of clinical indications for PET/CT studies in the three waves were comparable (p = 0.06). Conversely, the occurrence of patients positive for COVID-19 infection progressively increased (p < 0.0001) from the first to third wave; in particular, patients with COVID-19 had active infection before PET/CT study as confirmed by molecular oro/nasopharyngeal swab.

**Conclusion:**

Despite the restrictive medical measures for the emergency, the number of 2-[^18^F]FDG PET/CT studies was unchanged during the three waves guaranteeing the diagnostic performance of PET/CT imaging for oncological patients.

## 1 Introduction

A fundamental role of medical imaging techniques has been showed for the clinical management of patients with COVID-19 infection mainly to assess the pulmonary involvement, but also to evaluate different organs besides the lungs since the disease is characterized by multi-organ damage [1, 2]. The main clinical recommendation consists of performing computed tomography (CT) in case of severe respiratory symptoms considering the local prevalence of the disease; however, chest X-ray seems to be sufficient to diagnose the presence and extent of pulmonary opacifications even in patients with moderate symptoms [3]. Several previous studies reported the potential role of integrated positron emission tomography (PET) and CT with 2-deoxy-2-[^18^F]fluoro-D-glucose (2-[^18^F]FDG) to incidentally identify COVID-19 lung abnormalities in patients undergoing 2-[^18^F]FDG PET/CT for conventional oncological or non-oncological clinical indications [4, 5, 6]. Of note, these PET/CT imaging preliminary reports highlighted the clinical importance of an early detection of COVID-19 infection to guide subsequent patient management. In fact, oncological patients for which the wide majority of 2-[^18^F]FDG PET/CT studies is requested are at high-risk of COVID-19 infection [7]. Indeed, since incidental COVID-19 cases may occur in 2-[^18^F]FDG PET/CT scans, nuclear medicine physicians should be able to recognize typical patterns of the disease on the co-registered CT images; additionally, nuclear medicine staff should adopt all safety measures for preventing COVID-19 diffusion [8]. Moreover, the restrictive medical measures for COVID-19 emergency have implied subsequent limitations in imaging workflow, even though the diagnostic assistance for oncological patients should be guaranteed [9, 10]. We recently reported our experience regarding the diagnostic work-flow of 2-[^18^F]FDG PET/CT imaging during the first wave of the COVID-19 pandemic from February to April 2020 demonstrating an unchanged number of 2-[^18^F]FDG PET/CT oncological studies compared to the same period of the 2019 [11]. The present study was performed to compare the impact of COVID-19 pandemic on 2-[^18^F]FDG PET/CT imaging work-flow during the three waves in our medical institution of southern of Italy.

## 2 Material and methods

### 2.1 Patients

We retrospectively reviewed the results of 2-[^18^F]FDG PET/CT imaging studies acquired during the COVID-19 pandemic. In particular, the three periods representing the three COVID-19 waves were evaluated, each of three months, as follow: from February 3 to April 30, 2020 (first wave), from October 15, 2020, to January 15, 2021 (second wave) and from January 18 to April 16, 2021 (third wave). The number, the clinical indications and the imaging findings of 2-[^18^F]FDG PET/CT studies were directly compared. Before imaging study, patients were screened for COVID-19 infection using clinical assessment and/or laboratory evaluation by rapid serological tests and/or molecular oro/nasopharyngeal swabs (reverse transcription - Real Time PCR) according to the official guidance corresponding to the three different COVID-19 waves. In particular, in the first wave molecular oro/nasopharyngeal swabs were not available for all patients, but clinical screening for COVID-19 was performed in all cases; when COVID-19 infection was suspected according to clinical and/or imaging findings molecular oro/nasopharyngeal swab was performed.

### 2.2 PET/CT imaging

2-[^18^F]FDG PET/CT studies were acquired using a Gemini TF 64 scanner (Philips Healthcare, Best, The Netherlands). All patients fasted for at least 6 h prior to imaging, and blood glucose levels were <180 mg/dL at the time of tracer injection. PET scans were acquired in 3-D mode starting 60 min after 2-[^18^F]FDG administration (activity range 200–300 MBq, according to body weight). A low- (70 mA) and high-dose (230 mA) CT scans (rotation time 1.5 s, collimation 16 × 0.625) were acquired for attenuation correction of emission data. The sinogram of emission data was reconstructed using the 3-D row action maximum likelihood algorithm, considering attenuation, detector efficiency, scatter and random coincidence corrections. Attenuation correction was performed using CT images. CT and 2-[^18^F]FDG PET images were matched and fused into transverse, coronal and sagittal total-body images.

### 2.3 Image analysis

CT chest images were evaluated by two experienced radiologists who worked in consensus and reviewed each set-in random order to evaluate the presence and the location of abnormal findings in lung parenchyma according to COVID-19 reporting and data system (CO-RADS) classification [12]. In case of disagreement, a third senior radiologist was consulted to reach a final consensus for CT imaging interpretation. Successively, 2-[^18^F]FDG distribution in the lungs was qualitatively evaluated using PET-CT fusion images and maximum standardized uptake value (SUV max) was measured on areas of increased 2-[^18^F]FDG uptake corresponding to abnormal CT findings. SUV max value of the most 2-[^18^F]FDG -avid lung abnormality was recorded. In particular, CO-RADS classification of CT findings [12] represented the level of suspicion of COVID-19 infection graded as illustrated and listed in Table 1. Finally, the presence of 2-[^18^F]FDG abnormal uptake was also assessed on PET/CT total-body images in extra-pulmonary locations.

**Table 1:** CO-RADS classification on CT∗.

CO-RADS 0	Not interpretable: scan technically incomplete or of insufficient quality for artifacts
CO-RADS 1	With no suspicion: normal CT or non-infectious CT abnormalities
CO-RADS 2	Low suspicion: CT abnormalities consistent with infections other than COVID-19, absence of ground-glass opacities
CO-RADS 3	Indeterminate suspicion: uncertain CT findings for COVID-19 such as small unifocal, perihilar or homogeneous extensive ground-glass opacities
CO-RADS 4	High suspicion: unilateral peri-broncho vascular ground-glass CT opacities without any other typical findings
CO-RADS 5	Very high suspicion: typical bilateral multifocal ground-glass CT opacities with peripheral and/or basal distribution with or without parenchyma consolidations
CO-RADS 6	Proven very high suspicion: CO-RADS 5 with positive RT-PCR test for virus-specific nucleic acid

∗ Prokop M, van Everdingen W, van Rees Vellinga T, van Ufford JQ, Stöger L, Beenen L, et al. CO-RADS - A categorical CT assessment scheme for patients with suspected COVID-19: definition and evaluation. Radiology. 2020, 296:97–104.

### 2.4 Statistical analysis

Continuous data are expressed as mean ± standard deviation and categorical data as percentage. Two-tailed t test and chi-square test were used to compare the differences in continuous and categorical variables, respectively. A p value <0.05 was used to define statistical significance. Dummy variables were created to indicate diagnostic admissions that occurred during the three waves. Linear regression analysis was used to evaluate trends in the number of imaging studies over the periods. The Cochran-Armitage test was used to test for trend in the proportion of patients positive for COVID-19 infection referred for 2-[^18^F]FDG PET/CT evaluation over the three waves. Statistical analysis was performed with Stata 17 software (StataCorp, College Station, Texas USA).

### 2.5 Ethical approval

All procedures performed were in accordance with the ethical standards of the institutional research committee and with the principles of the 1964 Declaration of Helsinki and its later amendments.

### 2.6 Informed consent

Informed consent was obtained from all individual participants included in the study.

## 3 Results

A total of 861 2-[^18^F]FDG PET/CT imaging studies in 725 patients (388 men, mean age 64 ± 4 years) was acquired during the three waves of the COVID-19 pandemic. The majority (94%) of imaging studies (n = 812) was performed for diagnosis/staging (n = 300) or follow-up (n = 512) of neoplastic diseases. The remaining 49 (6%) studies were acquired for the evaluation of non-oncological diseases of which vasculitis (n = 10), endocarditis (n = 5), fever of unknown origin (n = 5), histiocytosis x (n = 2) or other inflammatory diseases (n = 27). In detail, the distribution of the number and the type of clinical indications for 2-[^18^F]FDG PET/CT studies in the three COVID-19 waves were comparable (Table 2). Conversely, the occurrence of patients positive for COVID-19 infection progressively increased (p < 0.0001) from the first (n = 0) to the second (n = 10) and the third (n = 21) waves (Table 2). In patients with COVID-19, the diagnosis of infection occurred before PET/CT study: 1.9 ± 0.9 months in the 10 patients of the second wave and 2.2 ± 0.9 months in the 21 patients of the third wave. All these patients had negative molecular oro/nasopharyngeal swab before imaging and in all cases PET/CT was performed for evaluation of oncological diseases.

**Table 2:** Number of 2-[^18^F]FDG PET/CT studies and COVID-19 positive patients during the three waves.

	Waves			p-value
	First	Second	Third	
Total PET/CT studies (n)	299	287	275	0.06
Oncological (n)	280	270	262	0.06
Non-oncological (n)	19	17	13	0.06
Patients with COVID-19 (n)	0	10	21	<0.0001

**Figure 1:**
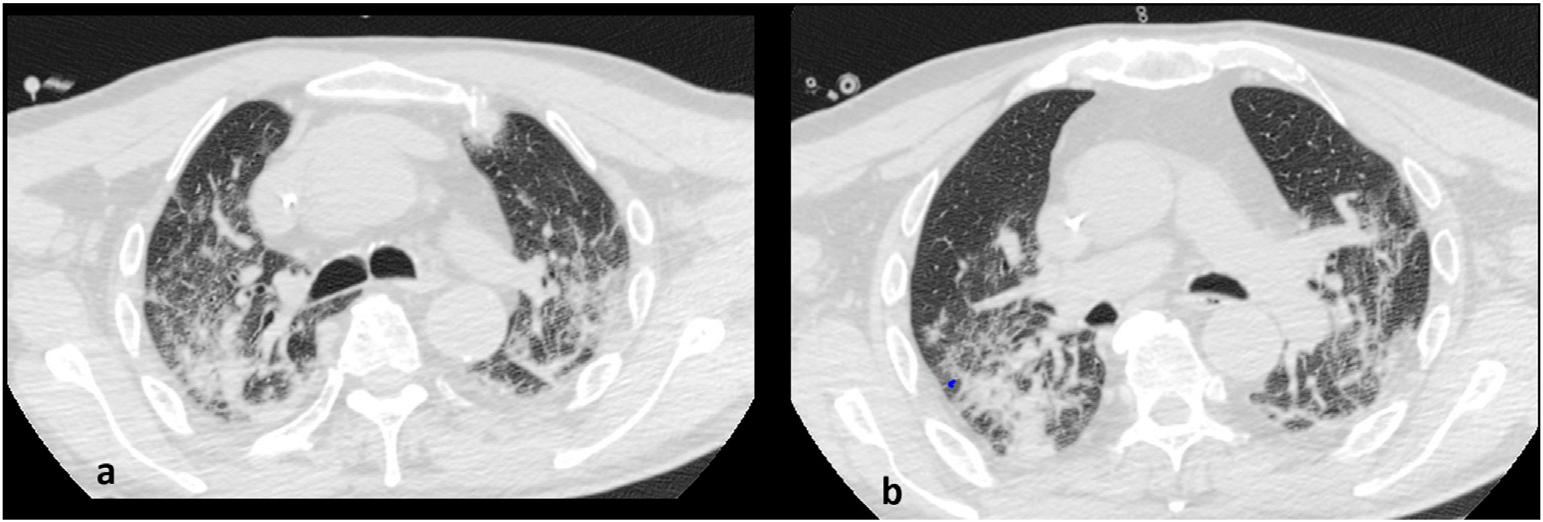
Chest CT (a and b) during active COVID-19 infection in a 68-year-old patient (#7 in Table 3) showing bilateral lung abnormalities (CO-RADS 6) suggestive of interstitial pneumoniae.

**Figure 2:**
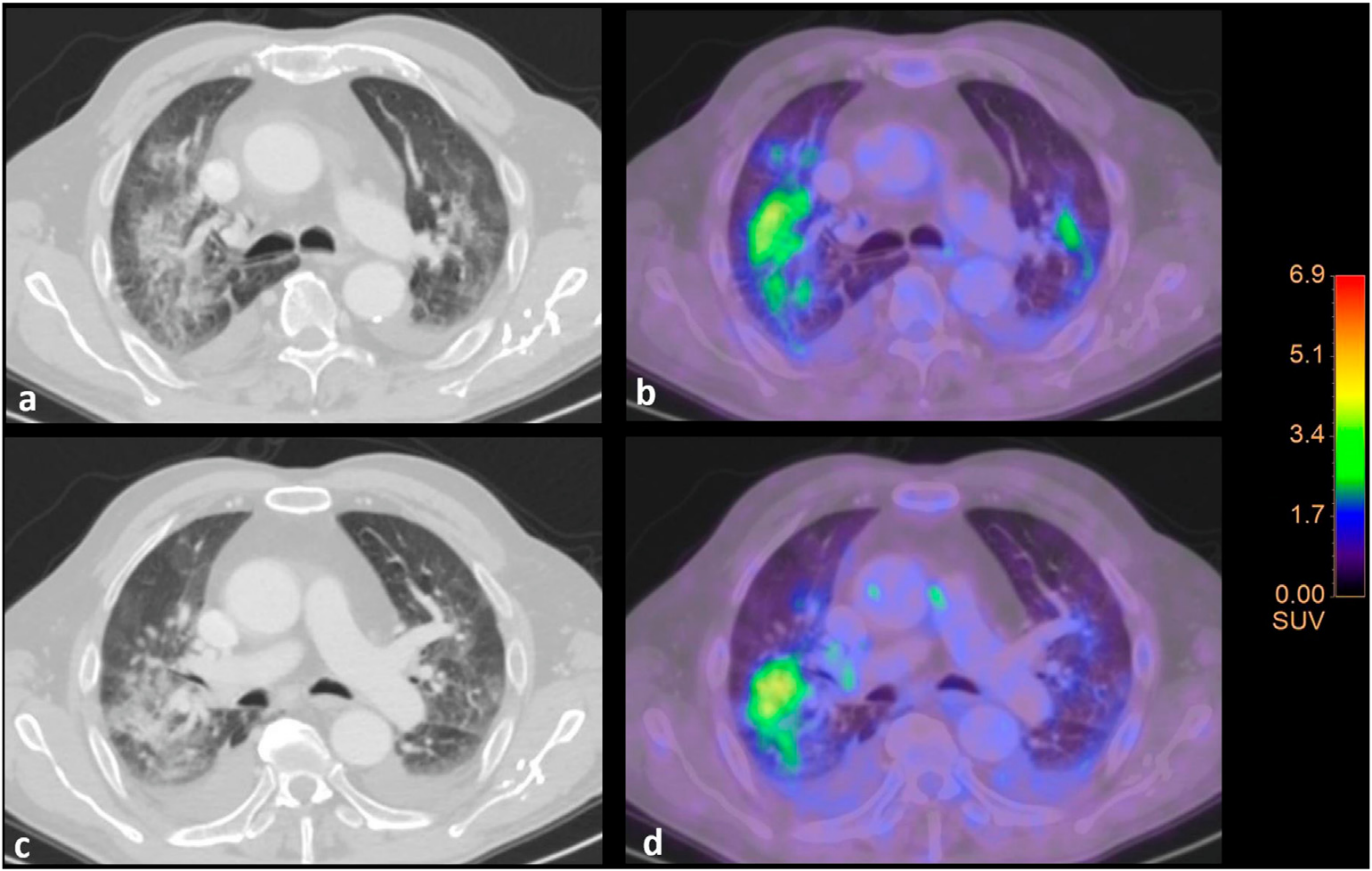
2-[^18^F]FDG PET/CT in patient of Figure 1 demonstrating diffused bilateral lung abnormalities (CO-RADS 5) (a and c) with increased inhomogeneous 2-[^18^F]FDG activity (SUV max 5.1) (b and d) as outcome imaging findings of interstitial pneumoniae by prior COVID-19 infection; bilateral pleural effusion was also present.

The clinical characteristics and PET/CT findings of the 10 patients positive for COVID-19 during the second wave are illustrated in Table 3. In this group no abnormal lung CT findings according to CO-RADS classification were observed in the majority (n = 8, 80%) of patients, while in the remaining two patients CO-RADS abnormalities were found of which only in one case the lung pattern was highly suspicious of COVID-19 infection (CO-RADS 5) with significant increase of 2-[^18^F]FDG uptake (SUV max 5.1); in the last patient a CO-RADS 2 pattern was observed with slightly increased 2-[^18^F]FDG uptake (SUV max 2.6). Figure 1 shows an example of a patient (#7 in Table 3) with respiratory symptomatic COVID-19 infection consisting of interstitial pneumoniae on CT with fever, cough, dyspnea and asthenia that was treated with medications and continuous positive airway pressure (CPAP). Two months later, after a negative molecular oro/nasopharyngeal swab, the patient underwent PET/CT to search a primary cancer of unknown origin (Figure 2).

**Table 3:** Clinical characteristics and 2-[^18^F]FDG PET/CT findings of patients with prior COVID-19 infection evaluated during the second wave.

#	Sex	Age (yr)	Tumor	Treatment	Imaging	COVID-19 infection		PET/CT lung findings		PET/CT extra-pulmonary localizations
						Symptoms	O_2_ therapy	CO-RADS	SUV max	
1	M	60	Esophageal cancer	Chemotherapy	Follow-up	Cough, asthenia	No	1	0	Neck and mediastinal LN
2	M	58	Melanoma of chest wall	Chemotherapy	Follow-up	None	No	1	0	Axillary, mediastinal and pelvis LN, prostate, bone
3	W	49	Rectal neuroendocrine tumor	Surgery, SSA-LAR	Follow-up	Cough, dyspnea	Yes	1	0	None
4	W	65	Non-Hodgkin lymphoma	Chemotherapy, BMT	Follow-up	Fever, asthenia	No	1	0	None
5	W	67	Breast and gastric cancer	Surgery, chemotherapy	Follow-up	Fever	No	1	0	Neck, mediastinal and abdominal LN, bone
6	W	84	Pancreatic cancer	None	Diagnosis	Fever, asthenia, dyspnea	Yes	1	0	Mediastinal and abdominal LN
7	M	68	Primary tumor of unknown origin	None	Diagnosis	Fever, cough, asthenia, dyspnea	Yes	5	5.1	Pericardial effusion, mediastinal and pelvis LN
8	M	36	Multiple myeloma	Chemotherapy	Follow-up	None	No	2	2.6	Bone
9	F	58	Hodgkin lymphoma	None	Diagnosis	Fever, asthenia, dyspnea	Yes	1	0	Neck, mediastinal and pelvis LN
10	W	68	Multiple myeloma	Chemotherapy	Follow-up	None	No	1	0	Neck LN, bone

SSA, somatostatin analogues; LAR, long-acting repeatable; BMT, bone marrow transplant; LN, lymph nodes.

**Figure 3:**
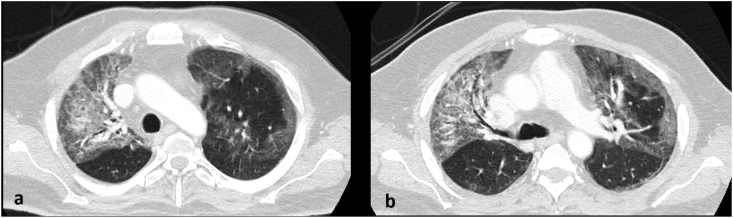
Chest CT (a and b) during active COVID-19 infection in a 58-year-old patient (#21 in Table 4) showing bilateral lung abnormalities (CO-RADS 6) suggestive of interstitial pneumoniae.

**Figure 4:**
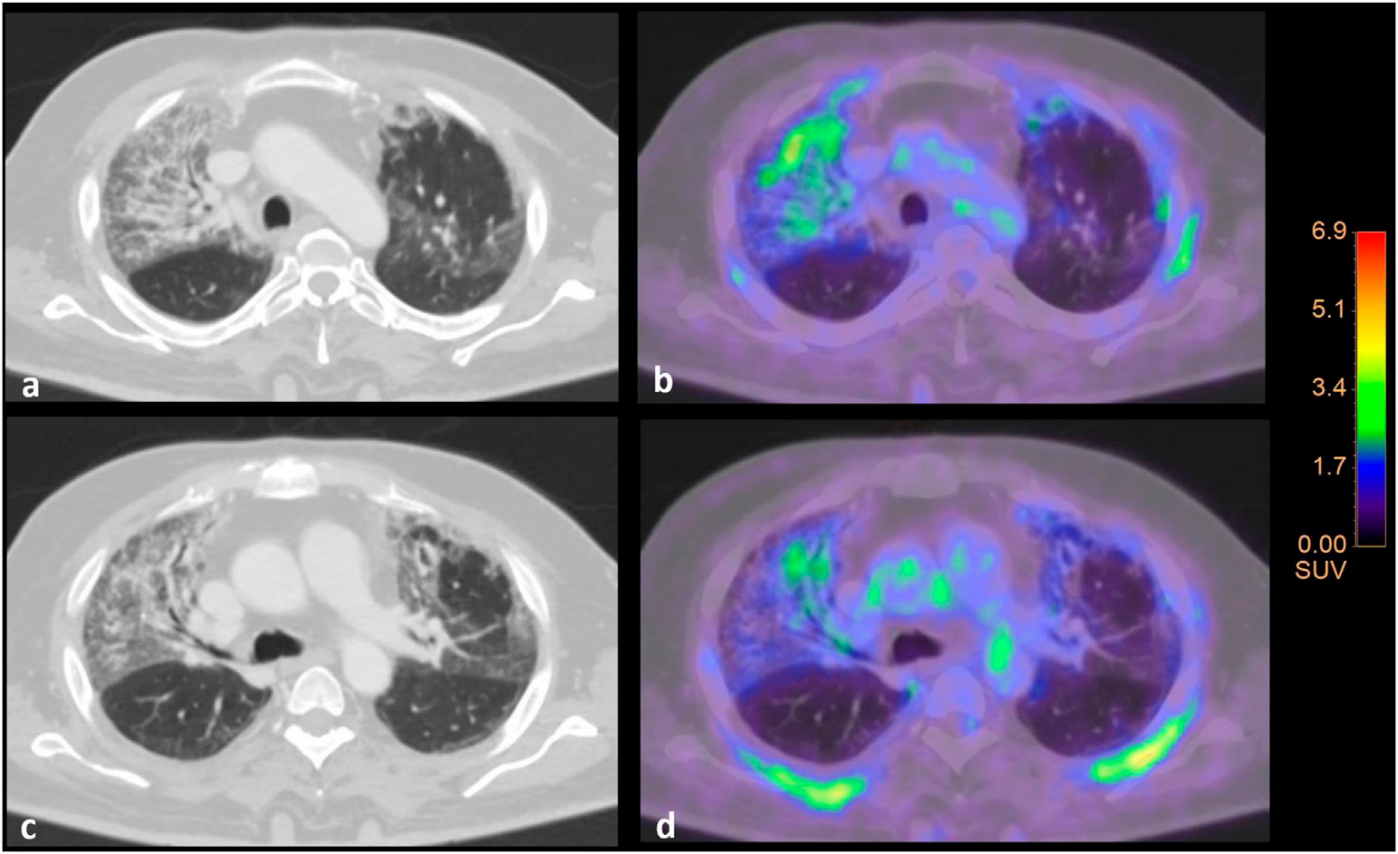
2-[^18^F]FDG PET/CT in patient of Figure 3 demonstrating diffused bilateral lung abnormalities (CO-RADS 5) (a and c) with increased inhomogeneous 2-[^18^F]FDG activity (SUV max 5.1) (b and d) as outcome imaging findings of interstitial pneumoniae by prior COVID-19 infection.

The clinical characteristics and PET/CT findings of the 21 patients positive for COVID-19 during the third wave are reported in Table 4. Also, in this group of patients no abnormal lung CT findings according to CO-RADS classification were observed in the majority (n = 16, 75%) of patients, while in the other five patients CO-RADS abnormalities were found of which in only one case the lung pattern was highly suspicious of COVID-19 infection (CO-RADS 5) with a significant increase of 2-[^18^F]FDG uptake (SUV max 5.1). In the remaining four patients CO-RADS lung patterns (3 or 2) were not highly suspicious for COVID-19 infection. Figure 3 shows an example of a patient (#21 in Table 4) with respiratory symptomatic COVID-19 infection consisting of interstitial pneumoniae on CT with fever, cough and dyspnea that was treated with medication and CPAP. Two months later the patient had a negative molecular oro/nasopharyngeal swab and underwent PET/CT to search a primary cancer of unknown origin (Figure 4).

**Table 4:** Clinical characteristics and 2-[^18^F]FDG PET/CT findings of patients with prior COVID-19 infection evaluated during the third wave.

#	Sex	Age (yr)	Tumor	Treatment	Imaging	COVID-19 infection		PET/CT lung findings		PET/CT extra-pulmonary localizations
						Symptoms	O_2_ therapy	CO-RADS	SUV max	
1	M	62	Multiple myeloma	Chemotherapy	Follow-up	Fever, cough, dyspnea	No	1	0	Bone
2	M	63	Colon cancer	Surgery	Follow-up	Fever, cough, asthenia, dyspnea	Yes	1	0	Neck, mediastinal and pelvis LN
3	W	81	Cholangiocarcinoma	None	Diagnosis	Asthenia	No	2	2.5	Neck, mediastinal and abdominal LN, bone
4	M	60	Colon cancer	Surgery, chemotherapy	Follow-up	Cough, asthenia	No	1	0	Neck, abdominal and pelvis LN
5	W	41	Rectal cancer	Chemotherapy	Follow-up	None	No	1	0	Liver, mediastinal LN
6	M	43	Thymus cancer	None	Diagnosis	Fever, cough, asthenia	No	1	0	Neck and inguinal LN
7	M	63	Gastrointestinal stromal tumor	Surgery, chemotherapy	Follow-up	Fever, cough, asthenia	No	1	0	Mediastinal and abdominal LN
8	W	53	Hodgkin lymphoma	Chemotherapy	Follow-up	Fever, dyspnea	Yes	1	0	Neck, mediastinal, abdominal and pelvis LN
9	W	51	Melanoma	None	Diagnosis	Fever, asthenia	No	1	0	Brain, soft tissues, neck and mediastinal LN
10	M	70	Multiple myeloma	None	Diagnosis	None	No	1	0	Neck, axillary, mediastinal, abdominal and pelvis LN, bone
11	W	58	Non-Hodgkin lymphoma	Chemotherapy	Follow-up	Fever, cough, asthenia, dyspnea	No	3	2.1	Stomach, mediastinal and abdominal LN
12	W	84	Pancreatic cancer	None	Diagnosis	Fever, asthenia, dyspnea	Yes	1	0	Mediastinal and abdominal LN
13	M	39	Lung cancer	None	Diagnosis	Fever	No	1	0	Neck, axillary, mediastinal and pelvis LN, spleen, bone
14	M	55	Melanoma	None	Follow-up	Fever, dyspnea	No	2	2.0	Soft tissue, abdominal and pelvis LN
15	W	60	Colon cancer	None	Diagnosis	Fever, cough, asthenia, dyspnea	No	1	0	Abdominal, mediastinal and pelvis LN
16	W	77	Colon cancer	Surgery	Follow-up	Fever, cough, dyspnea	No	1	0	Soft tissue, mediastinal LN
17	M	35	Primary tumor of unknown origin	None	Diagnosis	Fever, dyspnea	No	1	0	None
18	M	66	Colon cancer	Surgery, chemotherapy	Follow-up	Asthenia	No	1	0	Soft tissues, neck, axillary, mediastinal and abdominal LN
19	W	58	Hodgkin lymphoma	None	Diagnosis	Fever, asthenia, dyspnea	Yes	1	0	Abdominal and pelvis LN
20	W	71	Ovarian cancer	Surgery	Follow-up	cough, dyspnea	No	2	2.1	Neck, mediastinal, abdominal and pelvis LN
21	M	58	Primary tumor of unknown origin	None	Diagnosis	Fever, cough, dyspnea	Yes	5	5.1	Mediastinal LN, bone

LN, lymph nodes.

## 4 Discussion

Previous studies showed the potential role of PET/CT with 2-[^18^F]FDG to incidentally identify COVID-19 lung abnormalities in patients undergoing imaging for conventional, oncological or non-oncological, clinical indications [4, 5, 6]. In the present study, **we** compared the impact of COVID-19 pandemic on 2-[^18^F]FDG PET/CT work-flow during the three waves in our medical institution in the South of Italy. Our results indicate that, despite the restrictive medical measures for the emergency, the number of 2-[^18^F]FDG PET/CT studies was unchanged during the three waves guaranteeing the diagnostic performance of PET/CT imaging mainly (94%) for oncological patients; in detail, PET/CT studies during the three waves were performed not only for diagnosis/staging (n = 300) of tumor diseases, but mainly during the follow-up (n = 512). Furthermore, a progressive diffusion increase of COVID-19 infection was observed in the three waves, suggesting a major spread of pandemic in the South of Italy in the second and third wave.

In our experience, the majority of 2-[^18^F]FDG PET/CT studies acquired during each of the three waves was performed for oncological clinical indications and the number of imaging studies was comparable between the three waves. These findings reflect that during the emergency for pandemic the diagnostic assistance for neoplastic patients was guaranteed. While this pandemic triggered an immense pressure on the health-care system, the efforts to avoid any postponing in cancer diagnostics are of pivotal importance to avoid poorer outcomes in oncologic patients [7]. Moreover, we recently reported that the number of 2-[^18^F]FDG PET/CT oncological studies was unchanged during the first COVID-19 wave compared also to the same period of the previous year (2019) when there were not restrictive medical measures, furtherly supporting the diagnostic assistance for cancer patients in our institution [11]. A similar multi-center study was performed by Wong et al. [13] in England during the first COVID-19 wave comparing the impact of pandemic on oncological and non-oncological 2-[^18^F]FDG PET/CT workload. They found that the total number of 2-[^18^F]FDG PET/CT scans fell significantly during the first COVID-19 wave, with the reduction being greater for non-oncological PET/CT studies. These findings are at least partly due to the British Nuclear Medicine Society recommendation to prioritize patients with new diagnosis of cancer over other clinical indication for 2-[^18^F]FDG PET/CT scans [14]. However, other factors might have contributed to the decrease in 2-[^18^F]FDG PET/CT scans for oncologic patients, such as the decision of bypassing or delaying PET/CT scanning for safety reasons and/or patients' fear [15]. Similar results were reported in an international survey during the 2020 by Giammarile et al. [16] who showed that 2-[^18^F]FDG PET/CT imaging for oncologic indications showed a lesser decrease in utilization rates compared to conventional nuclear medicine procedures. Yet, Freudenberg et al. [17] in a national survey in Germany during the 2020 reported a decrease in conventional radionuclide studies, but a small (1.2%) increase in 2-[^18^F]FDG PET/CT examinations. Therefore, our experience and these other investigations confirm that 2-[^18^F]FDG PET-CT imaging for cancer patients was not significantly affected by the restrictive medical measures of COVID-19 pandemic. However, even though 2-[^18^F]FDG PET/CT imaging workflow for cancer patients was less impaired by the pandemic [11, 13], an overall reduction in nuclear medicine procedures has been reported in worldwide and national surveys [18, 19, 20]. Of note, the decrease and/or the postponement of diagnostic examinations due to the pandemic might impact on the disease's clinical course, potentially affecting the quality of life and patient survival [21]. Different factors might concur in the reduction of nuclear medicine departments activity, including patients' fears and preferences as well as safety measures adopted [20]. Therefore, efforts should be made towards balancing security measures and the need to ensure medical assistance without sacrificing patients' healthcare [21].

In our series, the 2-[^18^F]FDG PET/CT imaging evaluation of the 10 patients who had previous COVID-19 infection during the second wave demonstrated no significant lung abnormalities in the majority of patients of which 3 were asymptomatic, 3 had slight clinical symptoms, 3 had moderate respiratory symptoms requiring conventional O2-therapy, while only the last patient had severe respiratory symptoms needing CPAP treatment. Of note, only in this latter patient who had a severe respiratory failure a significant CO-RADS pattern suggestive of COVID-19 interstitial pneumoniae (CO-RADS 5) was observed as well as showing increased 2-[^18^F]FDG activity (SUV max 5.1). Similarly, the 2-[^18^F]FDG PET/CT imaging evaluation of the 21 patients who had previous COVID-19 infection during the third wave showed no significant lung abnormalities in the majority of patients of which 2 were asymptomatic, 14 had slight clinical symptoms, 4 had moderate respiratory symptoms requiring conventional O2-therapy, while only the last patient had severe respiratory symptoms needing CPAP treatment. Comparably, also in this patient with previous severe respiratory syndrome a significant CO-RADS pattern suggestive of COVID-19 interstitial pneumoniae (CO-RADS 5) was observed as well as showing increased 2-[^18^F]FDG activity (SUV max 5.1). In this regard, similar SUV values in lung abnormalities as CO-RADS 5 or 6 have been recently reported in a study by Wakfie-Corieh et al. [22] in which the correlation between metabolic and structural lung COVID-19 changes has been investigated. Our findings suggest that in patients with previous COVID-19 infection the presence of persistent lung abnormalities detected by 2-[^18^F]FDG PET/CT suggestive of interstitial pneumoniae may occur and seems to be related to previous severe clinical respiratory failure. This observation is in agreement with the results reported by Bai et al. [23] who described persistent lung 2-[^18^F]FDG PET/CT abnormalities in seven convalescing patients after severe COVID-19 infection and two consecutive negative molecular oro/nasopharyngeal swabs. A similar 2-[^18^F]FDG PET/CT finding was also described by Fu et al. [24] in single patient with previous COVID-19 infection. In this regard, a recent pathology report described interstitial mononuclear inflammatory infiltrates in both lungs of a patient with COVID-19 suggesting that significant inflammation may persist in the lungs during convalescence after the infection [25]. Thus, the increased 2-[^18^F]FDG uptake could be interpreted as reflecting increased glycolytic activity due to infiltration and inflammation of lung tissue.

In conclusion, this study shows that despite the restrictive medical measures for the COVID-19 emergency, the number of 2-[^18^F]FDG PET/CT imaging studies was unchanged during the three waves guaranteeing the diagnostic performance of PET/CT scanning mainly for oncological patients.

## Declarations

### Author contribution statement

Simone Maurea and Alberto Cuocolo: Conceived and designed the experiments; Performed the experiments; Analyzed and interpreted the data; Wrote the paper.

Claudia Bombace and Arnaldo Stanzione: Performed the experiments; Analyzed and interpreted the data; Wrote the paper.

Mario Petretta: Analyzed and interpreted the data; Wrote the paper.

Ciro Gabriele Mainolfi, Alessandra Annunziata, Ludovica Attanasio, Elide Matano, Brigitta Mucci, Alessandro D'Ambrosio and Claudia Giordano: Contributed reagents, materials, analysis tools or data.

Silvana Del Vecchio: Contributed reagents, materials, analysis tools or data; Wrote the paper.

### Funding statement

This research did not receive any specific grant from funding agencies in the public, commercial, or not-for-profit sectors.

### Data availability statement

The authors do not have permission to share data.

### Declaration of interests statement

The authors declare no conflict of interest.

### Additional information

No additional information is available for this paper.
